# Prediction of fatty acid composition in intact and minced fat of European autochthonous pigs breeds by near infrared spectroscopy

**DOI:** 10.1038/s41598-023-34996-x

**Published:** 2023-05-15

**Authors:** Silvia Parrini, Francesco Sirtori, Marjeta Čandek-Potokar, Rui Charneca, Alessandro Crovetti, Ivona Djurkin Kušec, Elena González Sanchez, Mercedes Maria Izquierdo Cebrian, Ana Haro Garcia, Danijel Karolyi, Benedicte Lebret, Alberto Ortiz, Nuria Panella-Riera, Matthias Petig, Preciosa Jesus da Costa Pires, David Tejerina, Violeta Razmaite, Chiara Aquilani, Riccardo Bozzi

**Affiliations:** 1 Department of Agriculture, Food, Environment and Forestry, University of Florence, Piazzale delle Cascine 18, 50144 Florence, Italy; 2 Kmetijski Inštitut Slovenije, Hacquetova ulica 17, 1000 Ljubljana, Slovenia; 3 MED – Mediterranean Institute for Agriculture, Environment and Development and CHANGE – Global Change and Sustainability Institute, Departamento de Zootecnia, Escola de Ciências e Tecnologia, Universidade de Évora, Pólo da Mitra, Ap. 94, 7006-554 Évora, Portugal; 4 Department for Animal Production and Biotechnology, Faculty of Agrobiotechnical Sciences Osijek, Vladimira Preloga 1, Osijek, Croatia; 5 Department of Animal Production and Food Science, School of Agricultural Engineering, University of Extremadura, Avda. Adolfo Suarez, s/n, 06007 Badajoz, Spain; 6 Centre of Scientific and Technological Research of Extremadura, CICYTEX, Badajoz, Spain; 7 Department of Nutrition and Sustainable Animal Production, Estacion Experimental del Zaidin, Spanish National Research Council, CSIC, Profesor Albareda 1, 18008 Granada, Spain; 8 Department of Animal Science, University of Zagreb Faculty of Agriculture, Svetosimunska cesta 25, 10000 Zagreb, Croatia; 9 PEGASE, INRAE, Institut Agro, 35590 Saint-Gilles, France; 10 IRTA-Monells, Finca Camps i Armet, s/n, 17121 Monells, Spain; 11 BESH, Haller Str. 20, 74549 Wolpertshausen, Germany; 12 Center for Research and Development in Agri-Food Systems and Sustainability (CISAS), Polytechnic Institute of Viana do Castelo. Praça General Barbosa, 4900-347 Viana do Castelo, Portugal; 13 Animal Science Institute, Lithuanian University of Health Sciences, 82317 Baisogala, Lithuania

**Keywords:** Biotechnology, Zoology

## Abstract

The fatty acids profile has been playing a decisive role in recent years, thanks to technological, sensory and health demands from producers and consumers. The application of NIRS technique on fat tissues, could lead to more efficient, practical, and economical in the quality control. The study aim was to assess the accuracy of Fourier Transformed Near Infrared Spectroscopy technique to determine fatty acids composition in fat of 12 European local pig breeds. A total of 439 spectra of backfat were collected both in intact and minced tissue and then were analyzed using gas chromatographic analysis. Predictive equations were developed using the 80% of samples for the calibration, followed by full cross validation, and the remaining 20% for the external validation test. NIRS analysis of minced samples allowed a better response for fatty acid families, n6 PUFA, it is promising both for n3 PUFA quantification and for the screening (high, low value) of the major fatty acids. Intact fat prediction, although with a lower predictive ability, seems suitable for PUFA and n6 PUFA while for other families allows only a discrimination between high and low values.

## Introduction

The proportions of fatty acids in pork fat took on a decisive role over the years, both due to the degree of fat unsaturation representing a key factor in the technological quality of processed meat and to the significant influence on qualitative parameters linked to sensory and nutritional profile^1^. High quality meat demand of consumers and processing industry is increasing, in particular meat and meat- products derived from local breeds has been growing interest due to the positive perception of their quality products in terms of health and animal welfare^2^. Fatty acids (FAs) composition of autochthonous breeds has been described as healthier and more responsive to the human nutritional requirements than the improved breeds by Prieto et al.^2^ and Lebret and Čandek-Potokar^3^. By nutritional viewpoint, individuals and classes of fatty acids are crucial in order to help consumers make healthy food choices as suggested by the European Parliament (EU Reg. 1169/2011) which added the obligation to report the total fat content and the saturated fat percentage as food information. Nevertheless, in autochthonous breeds the fresh and cured products mainly derived from small populations, which do not make the assurance of traceability and quality control technically and/or economically feasible^4^. Classical analysis to evaluate FAs profile has usually been performed by conventional “wet chemistry” procedures, such as gas chromatography, that need a lot of expertise, use of toxic reagents and it is very expensive and time consuming^5^. Spectroscopic techniques such as Near Infrared Spectroscopy (NIRS) can represent a resource for solving these problems thanks to its speed of analysis, simplicity, low environmental impact, and low operating cost. NIRS is based on the absorption of wavelengths or wavenumbers in the near infrared electromagnetic region determined by stretching and bending of atoms with specific chemical bonds and functional molecular groups. Fourier Transformation (FT-NIRS) represent a further advancement of NIRS technology improving signal-to-noise ratio in spectral resolution and wavenumber as well as environment features^6–8^. Furthermore, Lucarini et al.^9^ suggested that the Fourier Transformed application advantages include less or no sample preparation. NIRS technology is an indirect method and chemometrics analysis that include the use of multivariate regression^10^ to build up and develop the models comparing spectral results with samples of known composition. A big data set and a large number of samples are necessary to represent the full range of possible variability, especially when different variability factors, such as animal breed, are involved. A lack of robustness could be attributed to various sources of error, such as temperature and humidity of both the samples and analysis environment, differences between samples presentation, references, modes of analysis, etc.^11^. Furthermore, one of the main difficulties in the application of NIRS is the way in which the samples are presented to obtain representative NIR spectra. Avoiding sample milling is an advantage recognized by the pioneers of NIRS^12^. On the other hand, the possible non-homogeneity of the intact sample represents a limitation that directly affects the decrease in accuracy of the models compared to homogenized and minced samples presentation^13^. Nevertheless, the number of samples and their large variability are the main influencing factors that could complicate the accuracy of the NIRS results. In pig products, NIRS was initially used to predict the chemical composition of meat^13,14^, while more recently its application has led to more accurate analyses: online and offline rapid quality control of meat on a large scale^2,15,16^; physical and sensory characteristics^14,17–19^; control of the diets (growing or fattening period)^16,20^; prediction of defective or no defective classes, on the basis of pastiness and color^21^ and other sensory attributes^22^ in the case of Spanish ham. Considering that fatty acids structure could characterize fat spectral wavenumber, NIRS technology could be accessible for their detection. In the various studies, the characterization of the fatty acid profile, were focused on analysis of singular breed while few studies considered local as well as cosmopolitan breeds. Autochthonous breeds fatty acid determination by NIRS had been reported by González-Martín et al.^1^ and Caceres-Nevado et al.^15^, in fat and meat of Iberian pig respectively; Fernández-Cabanás et al.^23^ in Iberian pork dry-cured sausages; Prevolnik-Povše et al.^24^ in samples of meat and products originated by Krškopolje, and Turopolje breeds and Ortiz et al.^4^ in different European pig loin samples. Considering the importance of the direct application of NIRS to a whole slice (intact samples) instead of a minced sample, in the context of online determination, avoiding the destruction of product, and reducing the time required for analysis, only few studies have been carried out that considered different conditions for sample submission and preparation, e.g. comparing intact and minced samples. Caceres-Nevado et al.^15^ compared intact and minced loin samples for the prediction of chemical component while Zamora-Rojas et al.^16^ considered a classification on intact fat in the carcass and a skin-free subcutaneous fat sample from Iberian pigs fed with different regimes. For the studies on the prediction of FAs with NIRS, different methods of sample collection were considered: García-Rey et al.^25^ started from liquid fat, González-Martín et al.^1^ worked on intact subcutaneous fat of Iberian pigs, Pérez-Marín et al.^11^ applied NIRS directly on carcass, from subcutaneous fat sample with and without skin-free of Iberian pigs, Gjerlaug-Enger et al.^26^ used fat cut into small pieces (brick size: 3–5 mm) of Norwegian Landrace and Duroc pigs, and Zamora-Rojas et al.^27^ focused on fatty acid determination of intact adipose tissue from pure Iberian and Iberian-duroc crossbreds. Despite various studies, it is still difficult to characterize the fatty acid profile of pigs using NIR technology, especially when the samples come from local breeds that differ greatly among themselves and also within breeds. The development of NIRS assays for FAs monitoring could represent an interesting implementation in the quality control process, as well as its application on fat of different local breeds could be useful to provide a wider range of information to consumers interested in these niche products. In this context, the characteristics of meat and fat products from autochthonous pig breeds are linked to numerous factors such as rearing areas, production systems, feeding regimen, age of pigs but also from genetic features^28^ that consequently could affect the NIRS estimation capacity. The present study, which is part of a larger project on meat quality of autochthonous pig breeds, aims to evaluate the potential of FT-NIRS to predict fatty acid composition (individual and group) in fresh backfat of different European local pig breeds by comparing two types of samples NIR preparation spectra (intact and minced). The study could represent the key to challenge in the real application of spectroscopy techniques considering the multifactorial influencers such as the different local breeds.

## Results

### NIRS spectra characteristics

**Figure 1:**
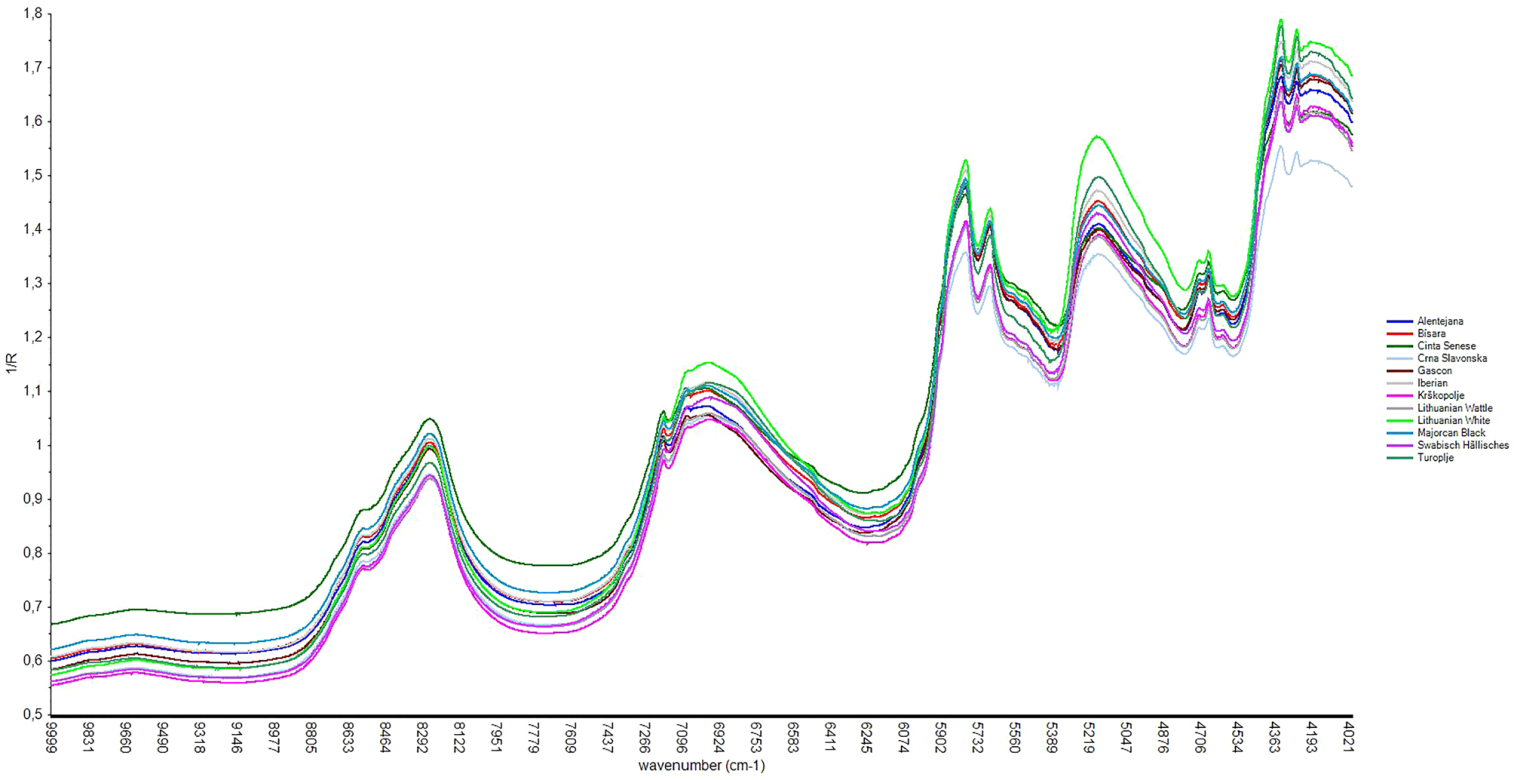
Average NIR spectra of intact samples of 12 studied breeds.

**Figure 2:**
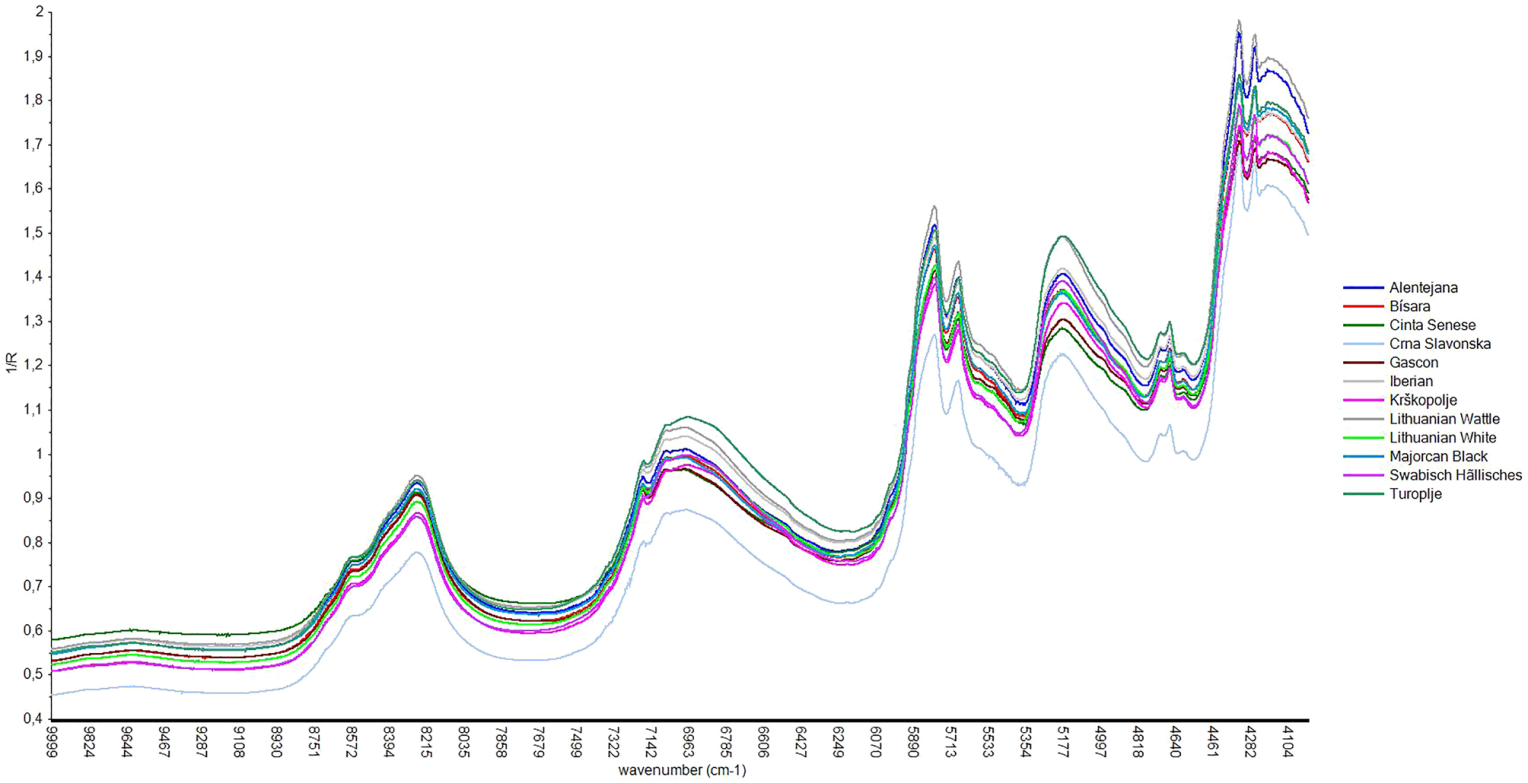
Average NIR spectra of minced samples of 12 studied breeds.

For each breed considered, the mean absorbance (log 1/R) derived from all the spectra of individual animals’ fat samples, intact and minced, were shown in Figs. 1 and 2, respectively. Breeds spectra showed similar trend and absorbance value among them both in intact and minced sampling, except for Crna Slavonska which showed a lower value along all wavenumbers. This similarity was also confirmed by directly comparing the two methods of presentation as average values of spectra derived from12 autochthonous pig and both presentation modes (Supplementary Fig. S1). As it has been noted, the shapes of the spectra are homogeneous and almost overlapping. However, as expected, the absorbance of intact samples was slightly higher than that of minced samples for almost all the wavenumbers.

In all breeds, the absorbance peaks between 5100–5200 cm^−1^, referred to the combinational vibration O–H stretching, could be associated with water^29^. Instead, N–H vibrational overtones, linked to protein content, were not evident at typical wavenumbers (4415, 5917, 6623, 8425 cm^–1^)^30^ probably because in the fat samples there was a low content of this constituent. The C–H absorption bands, characterizing the fat content, could be identified between 5700 and 5800 cm^−1^ which corresponds to the first overtone C–H stretching, between 8200 and 8500 cm^−1^, i.e. second overtone C–H stretching. Regarding fat, spectra also showed the peaks around 7100 cm^−1^, the consequential peaks between 4200 and 4400 cm^−1^, linked to combination of C–H stretching and finally, it is evident the consequential absorption peaks in the 4500–4600 cm^−1^.

### Descriptive statistics

Descriptive statistics were reported in Table 1 as for calibration and validation set. Fatty acid profile of samples showed a wide variability which may be associated with genetic and production system diversity that characterize each autochthonous breed. This variability, highlighted in both data set, is important in NIRS models, especially if the assessment of reliable and reproducible predictive ability by NIRS is tested of the whole variability.

**Table 1:** Descriptive statistics of fatty acid profile from data set used in calibration and validation (expressed as % of total identified fatty acids).

Parameter	Calibration				Validation			
	Mean	Min	Max	SD	Mean	Min	Max	SD
C12:0	0.088	0.049	0.131	0.015	0.087	0.049	0.130	0.016
C13:0	0.002	0.001	0.010	0.001	0.002	0.001	0.040	0.004
C14:0	1.492	1.041	2.316	0.212	1.503	1.068	2.316	0.228
C15:0	0.054	0.023	0.165	0.014	0.051	0.025	0.095	0.012
C16:0	25.666	18.784	31.167	2.193	25.846	20.606	30.243	2.236
C17:0	0.345	0.176	0.694	0.092	0.341	0.171	0.625	0.086
C18:0	13.052	8.644	20.384	1.855	13.260	9.088	20.229	2.090
C20:0	0.216	0.116	0.387	0.043	0.217	0.126	0.356	0.045
C22:0	0.013	0.002	0.085	0.013	0.012	0.003	0.052	0.010
Total SFA	40.950	29.687	51.733	3.589	41.337	32.284	51.141	3.781
C14:1	0.015	0.006	0.042	0.006	0.016	0.006	0.034	0.006
C16:1	2.578	1.566	4.621	0.595	2.583	1.566	4.261	0.622
C17:1	0.310	0.121	0.815	0.105	0.305	0.134	0.723	0.099
C18:1	45.303	36.335	55.434	3.698	45.307	37.883	55.055	3.592
C20:1	1.038	0.837	1.603	0.227	1.030	0.684	1.312	0.173
C22:1	0.023	0.006	0.088	0.019	0.026	0.007	0.080	0.021
Total MUFA	49.275	39.403	58.949	3.931	49.287	40.844	58.709	3.819
C18:2 n6	8.335	3.232	17.236	2.920	7.989	3.463	15.182	3.177
C18:3 n3	0.617	0.200	1.549	0.261	0.587	0.211	1.310	0.224
C20:2 n6	0.421	0.148	0.933	0.163	0.409	0.154	0.844	0.163
C20:3 n3	0.127	0.041	0.381	0.055	0.124	0.041	0.379	0.048
C20:3 n6	0.045	0.008	0.093	0.015	0.044	0.009	0.094	0.012
C20:4 n6	0.108	0.028	0.210	0.036	0.106	0.033	0.191	0.035
C20:5 n3	0.004	0.001	0.017	0.003	0.004	0.001	0.012	0.002
C22:4 n6	0.048	0.014	0.106	0.017	0.047	0.019	0.100	0.019
C22:5 n3	0.037	0.008	0.115	0.016	0.036	0.008	0.086	0.012
Total PUFA	9.773	3.765	20.484	3.380	9.376	4.025	17.507	3.690
n3 PUFA	0.787	0.271	1.975	0.320	0.752	0.283	1.708	0.288
n6 PUFA	8.956	3.456	18.483	3.115	8.594	3.715	16.150	3.409

*SD* standard deviation of mean, *Min* minimum, *Max* maximum.

### NIRS statistics results

The summaries of the statistics obtained from calibration, cross validation and external validation models in intact and minced samples were showed in Tables 2 and 3, respectively. For each parameter, range within NIR spectrum, optimal number of PLS factors and mathematical pre-treatment used was shown. Wavenumbers, selected in order to achieve best models, were reported for each parameter. Both for intact and minced samples a high number of PLS factors were necessary to develop the model. Standard normal variate followed by detrend as baseline correction resulted in the treatment that allowed to achieve the most accurate models. In addition, in intact samples, for some parameters it was necessary to apply a Savitzky–Golay polynomial filter (SG) to reduce the additive and multiplicative effects on spectral data^31^ even if when possible the lower number of pre-processing treatment were used. For almost all parameters, the best models were obtained taking into consideration specific regions of the near infrared spectrum. It seems that the length and the area of the near spectrum were linked to a group of fatty acids: individual and total SFA obtained the best model between 5400–7500 cm^−1^ region while total PUFA and relative fatty acids between 5400–6100 and 7400–8400 cm^−1^. Contrary, MUFA showed the best model considering the full spectrum of near a region slightly restricted in which only the tails have been cut (initial and final).

**Table 2:** Prediction statistics of fatty acid profile of intact fat.

Parameter	Spectrum range (cm^−1^)	Math. treat	nPLS	Calibration		Cross validation			External validation			
				R^2^	RMSE	R^2^cv	RMSEcv	RPDcv	R^2^v	RMSEv	RPDv	RER
C12:0	7500–5400	SNV; DT; SG	14	0.3262	0.0121	0.2402	0.0129	1.16	0.2150	0.0133	1.18	6.97
C13:0	7500–5400	SNV; DT	13	0.1505	0.0010	0.1042	0.0010	1.06	0.1040	0.0045	1.02	9.76
C14:0	7500–5400	SNV; DT; SG	13	0.3573	0.1683	0.2648	0.1806	1.18	0.1704	0.2059	1.11	6.53
C15:0	7500–5400	SNV; DT; SG	15	0.1501	0.0136	0.1170	0.0143	1.02	0.2167	0.0107	1.10	7.18
C16:0	7500–5400	SNV; DT	15	0.7204	1.1640	0.6617	1.2840	1.72	0.6820	1.2327	1.78	9.05
C17:0	7500–5400	SNV; DT	15	0.1970	0.0816	0.1080	0.0860	1.06	0.1014	0.0827	1.03	5.81
C18:0	7500–5400	SNV; DT	14	0.7351	0.9458	0.6927	1.0210	1.80	0.6970	1.0080	2.10	14.25
C20:0	7500–5400	SNV; DT	13	0.3013	0.0359	0.2332	0.0380	1.13	0.2622	0.0369	1.19	7.24
C22:0	7500–5400	SNV; DT	14	0.1590	0.0114	0.1500	0.0121	1.03	0.1490	0.0109	0.92	4.56
Total SFA	7500–5400	SNV; DT	15	0.8586	1.3512	0.8356	1.4610	2.46	0.8081	1.6320	2.29	14.24
C14:1	4500–9500	SNV; DT	13	0.3963	0.0045	0.3208	0.0047	1.24	0.3142	0.0040	1.46	6.79
C16:1	4500–9500	SNV; DT; SG	14	0.4649	0.4306	0.4018	0.4567	1.29	0.5258	0.4177	1.46	5.93
C17:1	4500–9500	SNV; DT; SG	15	0.3070	0.0881	0.2382	0.0927	1.14	0.3327	0.0825	1.23	7.38
C18:1	4500–9500	SNV; DT; SG	14	0.8744	1.3190	0.8517	1.4374	2.59	0.7714	1.6598	2.10	11.95
C20:1	4500–9500	SNV; DT; SG	14	0.3171	0.1710	0.2452	0.1807	1.15	0.2657	0.1747	1.17	6.71
C22:1 n9	4500–9500	SNV; DT; SG	14	0.3405	0.0153	0.2821	0.0160	1.18	0.3241	0.0181	1.20	4.34
Total MUFA	4500–9500	SNV; DT; SG	14	0.8853	1.3406	0.8660	1.4535	2.73	0.7812	1.7164	2.15	12.24
C18:2 n6	5400–6100/7400–8400	SNV; DT	13	0.9581	0.5963	0.9444	0.6892	4.23	0.9054	0.8876	3.26	14.63
C18:3 n3	5400–6100	SNV; DT	13	0.7988	0.1169	0.7411	0.1324	1.97	0.7988	0.1224	1.92	9.22
C20:2 n6	5400–6100/7400–8400	SNV; DT	14	0.7905	0.0740	0.7671	0.0790	2.07	0.7773	0.0808	2.13	8.74
C20:3 n3	5400–6100	SNV; DT	14	0.6257	0.3390	0.5553	0.0370	1.50	0.5441	0.0368	1.49	9.70
C20:3 n6	5400–6100/7400–8400	SNV; DT	14	0.5163	0.0104	0.4702	0.0109	1.38	0.5505	0.0101	1.51	8.37
C20:4 n6	5400–6100/7400–8400	SNV; DT	14	0.5752	0.0233	0.5364	0.0244	1.47	0.5817	0.0233	1.56	7.56
C20:5 n3	5400–6100	SNV; DT	14	0.4999	0.0017	0.4029	0.0018	1.35	0.3666	0.0019	1.26	7.46
C22:4 n6	5400–6100/7400–8400	SNV; DT	14	0.4262	0.0130	0.3782	0.0136	1.27	0.4358	0.0137	1.35	7.56
C22:5 n3	5400–6100	SNV; DT	14	0.5113	0.0100	0.4072	0.0118	1.30	0.5143	0.0107	1.45	7.70
Total PUFA	5400–6100	SNV; DT	14	0.9512	0.7450	0.9453	0.7913	4.27	0.9265	0.9071	3.70	16.36
n3 PUFA	5400–6100	SNV; DT	14	0.7967	0.1445	0.7312	0.1666	1.93	0.7256	0.1537	1.91	9.73
n6 PUFA	5400–6100/7400–8400	SNV; DT	14	0.9429	0.7424	0.9344	0.7983	3.90	0.9054	0.9522	3.26	14.41

*SNV* standard normal variate, *DT* de-trending, *SG* Savitzky–Golay filter, *nPLS* number of partial least square terms, *R*^*2*^*c* coefficient of determination in calibration, *RMSEC* root mean square error of calibration, *R*^*2*^*cv* coefficient of determination in cross-validation, *RMSEcv* root mean square error of cross validation, *RPDcv* residual prediction deviation in cross validation, *R*^*2*^*v* coefficient of determination of external validation, *RMSEv* root mean square error of external validation, *RPDv* residual prediction deviation in external validation, *RER* range error ratio in external validation, *SFA* saturated fatty acids, *MUFA* monounsaturated fatty acids, *PUFA* polyunsaturated fatty acids.

**Table 3:** Prediction statistics of fatty acid profile of minced fat.

Parameter	Spectrum range (cm^−1^)	Math. treat	nPLS	Calibration		Cross validation			External validation			
				R^2^	RMSE	R^2^cv	RMSEcv	RPDcv	R^2^v	RMSEv	RPDv	RER
C12:0	5400–7500	SNV; DT	14	0.4140	0.0100	0.3368	0.0123	1.23	0.4521	0.0115	1.37	6.86
C13:0	5400–7500	SNV; DT	13	0.1820	0.0009	0.1540	0.0010	1.09	0.1519	0.004	1.07	9.88
C14:0	5400–7500	SNV; DT	14	0.4856	0.1510	0.4160	0.1619	1.31	0.4639	0.167	1.37	7.70
C15:0	5400–7500	SNV; DT	15	0.2093	0.0125	0.1590	0.0130	1.09	0.1598	0.012	1.09	5.86
C16:0	5400–7500	SNV; DT	15	0.8055	0.9601	0.7689	1.0498	2.08	0.7987	1.020	2.23	9.05
C17:0	5400–7500	SNV; DT	12	0.2748	0.0792	0.1817	0.0844	1.10	0.1447	0.0865	1.01	5.49
C18:0	5400–7500	SNV; DT	14	0.8062	0.8332	0.7835	0.8706	2.15	0.7797	0.9667	2.13	9.67
C20:0	5400–7500	SNV; DT	14	0.3845	0.0336	0.3121	0.0352	1.20	0.4634	0.034	1.37	6.44
C22:0	5400–7500	SNV; DT	14	0.1923	0.0130	0.1570	0.0010	1.26	0.1994	0.009	1.17	6.07
Total SFA	5400–7500	SNV; DT	14	0.8971	1.1450	0.8789	1.2467	2.87	0.872	1.368	2.79	12.28
C14:1	4500–9500	SNV; DT	14	0.4000	0.0040	0.3704	0.0463	1.13	0.3628	0.0052	1.27	5.93
C16:1	4500–9500	SNV; DT	13	0.5690	0.4200	0.4510	0.4450	1.35	0.5090	0.4100	1.54	7.71
C17:1	4500–9500	SNV; DT	15	0.4080	0.0800	0.3440	0.0800	1.30	0.3628	0.0520	1.86	12.11
C18:1	4500–9500	SNV; DT	14	0.8980	1.1640	0.8745	1.2993	2.82	0.8789	1.2769	2.89	12.91
C20:1	4500–9500	SNV; DT	14	0.3733	0.1736	0.3704	0.1886	1.30	0.2798	0.1440	1.19	6.71
C22:1	4500–9500	SNV; DT	14	0.4080	0.0100	0.3470	0.0100	1.90	0.4307	0.0100	1.97	7.39
Total MUFA	4500–9500	SNV; DT	13	0.9056	1.1950	0.8976	1.2469	3.13	0.8875	1.3256	2.97	12.69
C18:2 n6	5400–6100/7400–8400	SNV; DT	13	0.9581	0.5963	0.9444	0.6892	4.24	0.9248	0.7989	3.63	14.71
C18:3n3	5400–6100	SNV; DT	13	0.8573	0.0982	0.8395	0.1043	2.51	0.7587	0.1260	2.04	9.38
C20:2 n6	5400–6100/7400–8400	SNV; DT	14	0.7980	0.0724	0.7600	0.0790	2.04	0.7450	0.0881	1.99	8.43
C20:3 n3	5400–6100	SNV; DT	14	0.6550	0.0315	0.6289	0.0327	1.65	0.6298	0.0327	1.84	10.85
C20:3 n6	5400–6100/ 7400–8400	SNV; DT	14	0.4928	0.0115	0.4620	0.0114	1.37	0.4164	0.0125	1.30	7.49
C20:4 n6	5400–6100/ 7400–8400	SNV; DT	14	0.6128	0.0220	0.5967	0.0229	1.58	0.5355	0.0245	1.47	6.45
C20:5 n3	5400–6100	SNV; DT	14	0.5559	0.0010	0.5214	0.0017	1.53	0.5214	0.0017	1.17	5.08
C22:5 n6	5400–6100/ 7400–8400	SNV; DT	14	0.4684	0.0125	0.4370	0.0129	1.34	0.3041	0.0134	1.21	5.05
C22:5 n3	5400–610/0	SNV; DT	14	0.5417	0.0100	0.5096	0.0109	1.44	0.5096	0.0109	1.42	7.51
Total PUFA	5400–6100	SNV; DT	13	0.9618	0.6590	0.9559	0.6796	4.98	0.9294	0.9004	3.75	15.11
n3 PUFA	5400–6100	SNV; DT	14	0.8604	0.1180	0.8406	0.1272	2.51	0.8990	0.1394	2.56	10.80
n6 PUFA	5400–6100/7400–8400	SNV; DT	14	0.9497	0.6975	0.9441	0.7362	4.23	0.9221	0.8718	3.57	14.36

*SNV* standard normal variate, *DT* de-trending, *nPLS* number of partial least square terms, *R*^*2*^*c* coefficient of determination in calibration, *RMSEC* root mean square error of calibration, *R*^*2*^*cv* coefficient of determination in cross-validation, *RMSEcv* root mean square error of cross validation, *RPDcv* residual prediction deviation in cross validation, *R*^*2*^*v* coefficient of determination of external validation, *RMSEv* root mean square error of external validation, *RPDv* residual prediction deviation in external validation, *RER* range error ratio in external validation, *SFA* saturated fatty acids, *MUFA* monounsaturated fatty acids, *PUFA* polyunsaturated fatty acids.

### Intact fat results

In intact samples, R^2^ coefficients were generally lower than those obtained in minced samples even if the pattern was similar with minimal differences, and close values in terms of R^2^ and errors for PUFA, SFA and MUFA families and some individual fatty acid as C18:2 n6, C18:1. Among SFA, R^2^ of the calibration for C16:0 and C18:0 was about 0.73, with value slightly lower in cross validation and external validation (R^2^ between 0.66 and 0.69). Other SFA fatty acids showed modest calibration R^2^ which was situated between 0.15 and 0.40 and always below 0.32 in both validation models. Total SFA gave the highest R^2^ which was 0.85, 0.83 and 0.80 respectively in calibration, cross validation, and external validation, respectively. With regard to MUFA fatty acids, the best results in terms of R^2^ was shown by C18.1 with R^2^ value of 0.87, 0.85 and 0.77 in calibration, cross-validation and external validation, respectively. For other individual MUFA fatty acids R^2^ was between 0.31 and 0.46 in calibration and between 0.24 and 0.52 in validation, with the lowest values reported for C20:1 indicating that the correlation was poor. Prediction model for the total MUFA content had R^2^ of 0.88, 0.86 and 0.78 in calibration, cross validation, and external validation, respectively. The best results for singular PUFA fatty acids in intact samples were achieved in calibration for C18:2 n6 (R^2^ 0.95), followed by C18:3 n3 (R^2^ 0.79) and C20:2 n6 (R^2^ 0.79) which reported R^2^ values in the range between 0.74 and 0.94 in cross validation and 0.77 and 0.90 in external validation. For other long-chain fatty acids (C20:3 n3, C20:3 n6, C20:4 n6, C20:5 n3, C22:4 n6, C22:4 n6, C22:5 n3) R^2^ reported values between 0.42 and 0.62 in calibration, and in any case values lower than 0.58 for validation. The highest R^2^ coefficients were obtained for total PUFA, 0.95, 0.94 and 0.92 in calibration, cross validation, and external validation, respectively. A high R^2^ values were also found for n6 PUFA group that achieved R^2^ of 0.94, 0.93 and 0.90 in calibration, cross validation, and external validation, respectively; contrary, the results for n3 PUFA were lower with a R^2^ from 0.72 to 0.79.

As regards RPD, which represents a ratio between RMSE of cross validation and SD, in the case of intact samples, cross validation values were between 1.5 and 2.5 for C16:0, C18:0, SFA, C18:3 n3, C20:2 n6, C20:3 n3, and n3 PUFA. Values higher than 2.5 were achieved for C18:1, SFA and MUFA, whileC18:2 n6, PUFA and n6 PUFA reached the best RPD ranging from 3.9 to 4.3. All the other fatty acids showed RPD values below 1.5 in cross validation. In the external validation, the RPDs were generally lower with values between 1.5 and 2.5 for C16:0, C18:0, SFA, C18:1, MUFA, C18:3 n3, C20:2 n6, C20:3 n6, C20:4 n6 and n3 PUFA. Also, in prediction the best RPDs were achieved by C18:2 n6, PUFA, n6 PUFA (values between 3.2 and 3.7).

Finally, the value RER (indicative of the suitability of models to categorize or quantify the samples), in models developed for intact samples showed values above 4, allowing a discrimination for all group or individual fatty acids. The RER limit of 9 was obtained by C13:0, C16:0, C18:0, C18:1, C18:2n6, C18:3 n3, C20:3 n3, SFA, MUFA, PUFA, n6 PUFA, n3 PUFA even if in the case of C13:0 it was linked to a very low R^2^ indicating that the model was not applicable.

Realistically, a RER above 10, linked both to an RPD close to 3 and a R^2^ > of 0.87 (in both validation models) was reported by C18:2 n6, PUFA and n6 PUFA while C18:1, SFA, MUFA. It thus seems easier to achieve more accurate models in cross validation losing accuracy in terms of RPD in external validation.

### Minced fat result

In minced samples among the SFA, C16:0, C18:0 showed the highest R^2^ of about 0.80 in calibration while in cross validation and external validation R^2^ was slightly lower and was between 0.76 and 0.79. The other SFA fatty acids showed modest R^2^ included between 0.18 and 0.48 in calibration and between 0.15 and 0.46 in both validation models. The sum of SFA presented a R^2^ of 0.89 and 0.87 respectively in calibration and validation. With regard to MUFA, the highest R^2^ was obtained for C18:1 achieving R^2^ of 0.89 in calibration and 0.87 in both validation models. The other MUFA showed models with R^2^ between 0.40 and 0.56 in calibration and of 0.34 and 0.50 in validation, except for C20:1 that exhibited a lower value. Total MUFA had calibration R^2^ of 0.90 and slightly lower values in cross and external validation (respectively 0.89 and 0.88). For individual PUFA, as in intact fat, the highest calibration R^2^ values were achieved in the case of C18:2 n6 (0.95), followed by C18:3 n3 (0.85) and C20:2 n6 (0.79), while in cross validation and external validation R^2^ ranged between 0.74 and 0.94. For other long-chain fatty acids (C20:3 n3, C20:3 n6, C20:4 n6, C20:5 n3, C22:4 n6, C22:4 n6, C22:5 n3) R^2^ was situated between 0.46 to 0.65 in calibration more without showing differences between n3 and n6 FA. Validation results, in terms of R^2^ were lower (0.62–0.32). The sum of PUFA gave the highest R^2^ in calibration (0.96), similar in cross validation (0.95) and slightly lower in external validation (0.92). For n6 PUFA group the achieved R^2^ was 0.95, 0.96 and 0.92 in calibration, cross validation, and external validation, respectively. Compared to that, the results for n3 PUFA group were lower (from 0.84 to 0.89).

As expected, root mean square errors were lower in calibration than in cross validation or external validation, while R^2^ value showed inverse pattern. The differences in root mean square error seem to depend on variability of the parameter considered: fatty acids with low concentration linked to less variability presented lower errors than higher values of the most abundant fatty acids.

Regarding the RPD in minced samples, values between 1.5 and 2.5 in cross validation were recorded in the case of C16:0, C18:0, C22:1, C20:2 n6, C20:3 n3, C20:4 n6 and C20:5 n3 even if in the case of C22:1 it was linked to a very low R^2^ indicating that the model was not applicable. The best result in terms of RPD in cross validation (higher than 2.5 or closed to 3) was obtained by SFA, C18:1, MUFA, C18:2 n6, C18:3 n3, PUFA, n3 PUFA and n6 PUFA. Considering external validation, RPD behavior was comparable to those obtained in cross validation, achieving values between 1.5 and 2.5 for the following fatty acids: C16:0, C18:0, C16:1, C17:1, C22:1, C18:3 n3, C20:2 n6 and C20:3 n3. Other parameters (SFA, C18:1, MUFA, C18:2 n6, PUFA, n3 PUFA and n6 PUFA) recorded higher values ranging from 2.8 to 3.8.

Finally, RER index in minced mode showed values above 4 for all parameters suggesting a discrimination capacity for all parameters. Certain fatty acids or groups (C13:0, C16:0, C17:1, C18:0, C18:1, C18:2n6, C18:3 n3, C20:3 n3, SFA, MUFA PUFA, PUFA n6, PUFA n3) reached RER values higher than 9, even if in the case of C13:0, C17:1 and C20:3n3 they were not linked to an adequate RPD or R^2^. Effectively, a RER above 10, linked both to an RPD close to 3 and a R^2^ > of 0.8 (in both validation models) was obtained for C18:1 C18:2 n6, SFA, MUFA, PUFA and n6 PUFA. In addition, both n3 PUFA and C18:3 n3 in minced samples highlighted better results than in intact ones in terms of closeness to limit of values necessary for the model’s application.

## Discussion

The features of the spectra, belonging to the NIR region, are represented by the absorption produced by the combination of harmonics and overtones of the fundamental frequencies of the functional groups. Visual identification is suggested in NIRS studies in order to detect the presence of compounds and to reduce the spectral region from which to extract the useful information, even if the recognition of the individual chemical compounds is not always accessible. Moreover, in the studied NIR spectra, the visual evidence of the main tissue constituents (moisture/water, fat and protein) was confirmed but the characteristic bands showed a slight shift along the wavenumber axis depending on the type of samples or instrument^32,33^.

The absorption bands of water always found in biological samples could easily be distinguished thanks to the presence of first stretch overtones and the valley absorption curve at 5500–6200 cm^−1^ that follows the first harmonic transitions of C–H bonds^33,34^. Typical fat spectra bands were evident around the characteristic wavenumber according to previous study on meat^32,35,36^. The consequential peaks at 5670 and 5800 cm^−1^ could be indicative of cis double bonds of unsaturated fatty acids according to Pieszczek et al.^36^ and Garrido-Varo et al.^37^. As for proteins, the very low content in the fat samples, taking the 5% limit proposed by ElMasry et al.^33^ as a reference, represent a modest contribution to the characteristics of the spectra. Furthermore, the protein absorption was likely masked by the strong peak of water and fatty acids in the same wavenumber regions as reported by Tsai et al.^38^ and ElMasry and Nakauchi^33^. The development of predictive models showed that the best relationships were obtained in a specific region of the near infrared spectrum. In almost all cases, the importance of selecting a specific spectral region was confirmed within each group of fatty acids. In agreement with our results, different studies on pig loin^4,32,39^ reported the best predictive models with the selected spectral range. In contrast, other studies^15,40^ suggested that the optimization of spectral regions demands more processing time than considerable improvements. In particular, Cáceres-Nevado et al.^15^ comparing full spectrum and a selected range didn’t achieve statistically significant difference in calibration approaches. In all cases, pretreatment of the spectra, SNV and DT proved useful to remove the effects of scattering and reduce the multicollinearity. In addition, the confounding effects of baseline shift and curvature were likely reduced due to spectral difference calculations^1^. However, the lower number of math pre-processing treatments on spectra was always considered in order to avoid the complexity of interpretation, the loss of some information and the minor structural differences among very similar signal profiles^41^ In various autochthonous breeds used in this research, spectral behavior was similar, exception being Crna Slavonska where the spectral discrepancy in absorbance occurred in minced samples. The difference in absorption capacities between intact and minced samples was consistent with previous studies on meat^4,15^ which reported the effect of the structural loss of tissue. Cozzolino et al.^42^ and Fan et al.^43^, working on lamb and pork muscles, respectively suggested that grinding interferes with structure of muscles thus affecting light absorbance. The wide variability in fatty acids values, especially for the fatty acids present in greater quantities was related to the diverse production systems and diets to which the different local pig breeds are subjected in their respective farms and countries^44^. This variability could be useful and positive for the development of predictive models by NIRS technology^45,46^. As expected, and as mentioned by other authors^15,43^ the best coefficients of determination for cross-validation and external validation were observed in the minced presentation mode, even if the trend of the results was the same. Sample preparation conditions are recognized as one of the key factors influencing the capacities of the NIRS and it is well known that homogenization improves the accuracy of NIRS^26^. However, mincing the fat is time consuming and could be difficult to homogenize with a mixer because the composition changes and tissue components attaching to the equipment can create errors^26^. The comparison with other studies in NIRS research is always difficult due to the different instruments (size of irradiated surface, signal/noise ratio, depth of light penetration), chemometrics models, parameters used and environmental conditions present^39^. The NIRS research developed for the prediction of fatty acids were more abundant and spread for minced samples than for intact samples. However also in the case of minced samples often studies considered only one breed (Iberian pig samples) obtaining statistics in validation similar or slightly higher for R^2^ to present work, but moderately lower errors^27^ probably due to the different set sizes. Prevolnik Povše^24^, who studied fat of two local breeds (Slovenian Krškopolje pig and Croatian Turopolje pig) reported similar or slightly lower cross validation R^2^ for SFA, MUFA and PUFA group, while the cross-validation errors slightly higher in our research. In the study by Müller et al.^40^ on pork fat from different carcass batches, a similar R^2^ was found in the prediction for MUFA, PUFA, C18:1 and C18:3 n3 while lower R^2^ were obtained for C18:2 n6 and higher for SFA and C18:0. Also in this case, the errors in prediction presented were lower compared to those obtained by our models except for C18:3 n3. Previous study on melted fat obtained better results in all cases than in the present study^25,26,47^ with errors of cross-validation or prediction ranging from 0.26 to 0.87 for C16:0, from 0.27 to 0.64 for C18:0, from 0.20 to 0.59 for C18:1 and from 0.15 to 0.36 for linoleic acid. Those results were probably linked to the melting condition that can affect precision and accuracy in the results. Also, Flåtten et al.^48^ reported that in purified fat better results were achieved, even if in his study LC PUFA were predicted by mid-infrared transmission. Regarding the NIRS results of prediction (external validation), the obtained values were in the same order as those obtained with the cross validation confirming the goodness of the proposed models of the present work. Even if the higher number of samples considered in our study positively affected the applicability of NIRS, the relevant number of factors variability involved within samples sets (diet, rearing systems, etc.) have probably affected accuracy and precision of estimation statistics with direct effects on errors. Moreover, in our study variability of each breed was directly affected by different traditional breeding conditions of each country.

Considering the main objective of this work, the evaluation of the NIRS method for simultaneous measurement of fatty acid composition in back fat of different autochthonous pig breeds, the RPD and RER indexes used to evaluate the capacity of the models suggested that the NIR equation of C18:1, C18:2 n6, SFA, MUFA, PUFA and n6 PUFA could be considered usable in most applications, including quality starting from minced fat samples. Promising results were also obtained for the quantification of C18:3n3 and n3 PUFA. For some of the major constituents (C16:0, C18:0, C20:2 n6, C20:3 n3), the RPD achieved, linked to a RER > 9 and to a R^2^ > 0.62 allowed for discrimination to differentiate high, medium and low values that could be useful on quality control categorization^49^. According to Müller et al.^40^, the calibration of minor fatty acids resulted generally poorer in terms of R^2^, RMSE, RPD and RER suggesting that NIRS cannot be used to quantify all individual fatty acids simultaneously although minced samples were used. In addition, as reported by Gjerlaug-Enger et al.^26^ NIRS has the best predictive ability for organic components with large volumes. However, both RPD and RER as well as R^2^ are highly dependent on the range of values in the calibration. Finally, even if RPD statistic is widely used in NIRS research for assessing the predictions efficiency^50^, Cáceres-Nevado et al.^15^ suggested that this criterion cannot be generalized to all types of products or all NIRS instruments.

Considering the results obtained for intact fat, Pérez-Marín et al.^39^ working on skin-free subcutaneous intact fat of Iberian pig on cross validation, reported higher R^2^ than our results for C16:0 (0.88), C18:0 (0.80) and C18:1 (0.92) and lower error on average. González-Martin et al.^1^ on subcutaneous fat of Iberian pigs achieved in external validation both slightly higher results of R^2^ and lower errors for C16:0, C18:0 and C18:1. On the contrary, the coefficients of determination of C18:2 and especially C18:3 n3 are higher in our study than in the mentioned research. Also, Pérez-Marín et al.^39^ reported calibration models poorer than our study for C18:2 (R^2^ 0.42) connecting these results with a lower variation data set as shown by the standard deviation of 1/3 than ours (0.75 vs. 3.0%). Minor fatty acids are rarely reported in that research and the results are often inconsistent: González-Martín et al.^1^ reported higher R^2^ than ours for C14:0 and lower values for C20:1.

Regarding the fatty acid group, in external validation González-Martín et al.^1^ achieved lower R^2^ for MUFA and PUFA, and higher R^2^ and lower error for PUFA. However, it must be noted that the variation of fatty acids for Iberian pigs^1,39^ was generally lower than those considered in our research because in Spain pigs are fed several diets but on the basis of the same extensive feeding programs and similar strategies. Gjerlaug-Enger et al.^26^, working on fat layers from Norwegian Landrace and Duroc pigs cut into small pieces (brick size: 3–5 mm), obtained slightly higher results for R^2^, RPD and RER for both the group and individual fatty acids. Nevertheless, a relative variation was considered by Gjerlaug-Enger et al.^26^ study because calibrations were made starting from pigs fed almost the same diet and tested in two experimental stations. It is stressed that in NIRS prediction the variation should cover the population in which the calibrations will be used for subsequent predictions: a larger variability in fatty acids could be obtained if pigs came randomly from different rearing system even if the NIRS capacity and accuracy can tend to decrease^26^. However, Prieto et al.^46^ suggested the use of specific prediction equations within each breed, as breed differences in NIRS meat fatty acid estimation were determined in finished animals fed a similar dietary regime and sourced from a single experimental farm. These authors reported genetic differences between breeds as the most influential factor in the accuracy of fatty acid estimation, that could be also associated to a different size of the adipocytes between the breeds linked to the absorbance in the collection of NIR spectra. At the current state, more research is needed to validate the patterns and results of NIRS estimation within different breeds.

In order to consider the model suitability for estimation of fatty acids from intact fat, the simultaneous consideration of the coefficient R^2^, RPD and RER indicates that the model was efficient for the practical quantification application of C18:2 n6, PUFA and n6 PUFA. The SFA and MUFA group, as well as C18:1 models could be considered to be suitable for screening purposes^50–52^. Finally, the possibility to categorize sample discriminating between high and low fatty acids values with acceptable precision seem to be promising for C16:0, C18:0, C18:3 n3 as well as C20:2 n6 and n3 PUFA.

## Methods

### Ethics approval and consent to participate

Animal Care and Use Committee approval was not necessary because backfat samples were collected after slaughtering of animals. The authors did not have direct control over the care of the animals because the experimentation of this study did not include the analysis of the subjects' life stages.

### Sample collection

A total of 439 backfat samples were collected after slaughter from subjects belonging to 12 European local pig, in the frame of H2020 project TREASURE (Table 4).

**Table 4:** Breeds and total number of backfat samples and origin country used in the study.

Breed	Total number of backfat samples	Origin country
Alentejana	19	Portugal
Bísara	54	Portugal
Cinta Senese	19	Italy
Crna Slavonska	16	Croatia
Gascon	76	France
Iberian	75	Spain
Krškopolje	35	Slovenija
Lithuanian Wattle	5	Lithuania
Lithuanian White	23	Lithuania
Majorcan Black	40	Spain
Schwabisch Hãllisches	57	Germany
Turopolje	20	Croatia

Subcutaneous fat (backfat) was sampled 1–2 days after slaughter from the left half-carcasses between the second to the fifth lumbar vertebra, individually vacuum packed and frozen at − 20 °C and sent to the University of Florence laboratory. After thawing, intact fat samples were scanned by FT-NIRS. Subsequently, samples were minced by electric meat grinder and scanned by FT-NIRS. Once the scans were acquired, the same samples were further used for gas chromatographic analysis. For each animal, all analysis were performed in duplicate.

### Reference analysis

Total lipids content was determined using the method of Folch et al.^53^; fatty acid profile of total lipids, using the modified technique of Morrison and Smith^54^.Fatty acids (FAs) methyl esters were analyzed by gas chromatography using a Varian 430 apparatus (Varian Inc., Palo Alto, CA, USA) equipped with a flame ionisation detector. FAs separation occurred in a Supelco Omegawax TM 320 capillary column (30-mlength; 0.32 mm internal diameter; 0.25 lm film thick-ness; Supelco, Bellafonte, PA, USA). The chromato-graphic conditions were an initial temperature of 160 C, which was then increased by 2 C/min until the temperature reached 220 C. One microliter of sample in hexane was injected with the carrier gas (helium) at a constant flow of 1.5 mL min^−1^ and at a split ratio of 1:20. The detector temperature was set at 260 C. The chromatograms were recorded using computing integrator software (Galaxie Chromatography Data System 1.9.302.952; Varian Inc.). The percentage of each fatty acid was calculated on the total of fatty acids detected and expressed as g/100 g of FAMEs. Fatty acid groups were obtained as sum of all saturated fatty acids (SFA) detected, sum of all monounsaturated fatty acids (MUFA) and sum of all polyunsaturated fatty acids (PUFA).

### FT-NIRS data pre-treatment and chemometric analysis

Spectra were processed by chemometric approach using Unscrambler CAMO^®^ software. To optimize the accuracy of calibration, several mathematical pre-treatments were applied: multiplicative scatter correction (MSC) and standard normal variate (SNV), with or without the de-trending (DT) option were applied for the correction of scatter effects in the spectra, spectral derivative Savitzky–Golay polynomial filter (SG) including a smoothing step before derivation (with 10 smoothing left side points and 9 smoothing right side points) avoiding reduction of the signal to noise ratio were applied when necessary. Furthermore, to optimize the extraction of useful information a selection or reduction from spectra were applied analyzing the spectra at the specific wavenumbers. Outliers were detected by both observing spectra line plot and principal component analysis (PCA) results. Possible outliers were identified as samples with high residual values and high Hotelling’s T^2^ statistic referred to spectra range (T > 2.5 as often reported for the removal of outliers)^55^. A scatterplot of leverage, respectively for intact (Supplementary Fig. S2) and minced (Supplementary Fig. S3) samples, were also considered in order to detect outliers. The obtained data set was split in two stratified data sets: a training (calibration) set with 80% of the samples and a validation set including the remaining 20% of the samples. In both sets, however, all breeds were included, guaranteeing the presence of 20% of animals for each breed in validation set. All models were built using partial least square regression (PLS), after other models as principal component regression (PCR) were evaluated and discarded because of lower predictive ability. To develop the model, for each parameter, the optimum number of PLS factors (nPLS) has been selected based on the one that determined the lowest error in cross validation and thus avoiding overfitting. Indeed, an internal cross-validation using the leave one-out method was applied on the training set and both the coefficient of determination of cross validation (R^2^cv) and root mean square errors in cross validation (RMEcv) were obtained. All calibrations were evaluated on the basis of both the entire NIR spectrum and specific regions, considering previous studies and the band/overtone present in our spectral data set^32,35,36^. The best model for each trait was evaluated based on the highest coefficient of determination in calibration (R^2^) and in external validation or prediction (R^2^v) as well as on the lowest root mean square error in calibration (RMSE) and prediction (RMSEv).

Residual prediction deviation (RPD) index was calculated as standard deviation (SD) of the set of samples and the RMSE ratio in cross validation (RPDcv) and in external validation (RPDv), in order to evaluate goodness of fit and model accuracy. The relationship between the interval of composition of the reference data for the collective calibration (Ymax − Ymin) and the RMSEv, known as the range error ratio (RER) index, was calculated as statistics indicators of the greatest weight in the precision of a NIRS calibration model^50^. The model performance can be considered sufficient for a rough screening if RPD is between 1.5 and 2.5^52^. Williams and Sobering^51^ suggested an ‘accurate estimation capacity’ if RPD values were higher than the limit of 2.5, even though afterwards the limit for the accuracy evaluation was increased to 3^52^, because the error of prediction is reduced by a factor of more than three^56^. A RER between 4 and 8 suggests the possibility of discriminating high values from low ones, while RER values in the range of 8–10 represent the possibility of predicting quantitative data and an RER above 10 or 12 indicates good predictability^5,49^.

## Conclusion

In conclusion it seems possible to use NIRS technology for the prediction of principal fatty acid families (SFA; MUFA and PUFA as well as n6 PUFA) and some singular fatty acid as C18:1 and C18:2 n6 coming from a large population of European autochthonous pigs’ breeds if minced fat samples are used. The homogenization of fat is promising for the quantification of C18:3 n3 and n3 PUFA and allow the screening (high and low value) for some major important constituents (C16:0, C18:0, C20:2 n6, C20:3 n3,) while it seems to be more difficult for other fatty acids.

Prediction on intact fat samples, although displaying lower predictive ability, has the advantage of being instantaneous and could be applied on marketable products. It seems suitable for PUFA and n6 PUFA as well as for C18:2 n6 while for other families (SFA and MUFA) as well as for C18:1 a discrimination between high and low values would be feasible.

The study of the specific wavenumbers at which NIR are closely associated with the fatty acid group composition resulted useful in order to achieve accurate calibrations. Moreover, the large variability of fatty acids used in this study could have affected the robustness of models. NIR spectroscopy will become more widely used in quality control, industries or breeding programs as more attention is given to reduce errors.

## Supplementary Information

Supplementary Information 1.

Supplementary Information 2.

Supplementary Information 3.

## Data Availability

The datasets used and/or analyzed during the current study are available from the corresponding author on reasonable request.

## References

[1] González-Martín I, González-Pérez C, Hernández-Méndez J, Alvarez-García N (2003). Determination of fatty acids in the subcutaneous fat of Iberian breed swine by near infrared spectroscopy (NIRS) with a fibre-optic probe. Meat Sci..

[2] Prieto N, Pawluczyk O, Dugan MER, Aalhus JL (2017). A review of the principles and applications of near-infrared spectroscopy to characterize meat, fat, and meat products. Appl. Spectrosc..

[3] Lebret B, Čandek-Potokar M (2022). Review: Pork quality attributes from farm to fork. Part I. Carcass and fresh meat. Animal.

[4] Ortiz A (2020). Potential use of near-infrared spectroscopy to predict fatty acid profile of meat from different European autochthonous pig breeds. Appl. Sci..

[5] Barbin DF (2015). Prediction of chicken quality attributes by near infrared spectroscopy. Food Chem..

[6] Aparicio Tovar MA, Vargas Giraldo JD (2006). Considerations on ethics and animal welfare in extensive pig production: Breeding and fattening Iberian pigs. Livestock Sci..

[7] Temple D, Manteca X, Velarde A, Dalmau A (2011). Assessment of animal welfare through behavioural parameters in Iberian pigs in intensive and extensive conditions. Appl. Anim. Behav. Sci..

[8] Vitale M (2020). Consumers’ expectations and liking of traditional and innovative pork products from European autochthonous pig breeds. Meat Sci..

[9] Lucarini M, Durazzo A, Sánchez del Pulgar J, Gabrielli P, Lombardi-Boccia G (2018). Determination of fatty acid content in meat and meat products: The FTIR-ATR approach. Food Chem..

[10] Kumar N, Bansal A, Sarma GS, Rawal RK (2014). Chemometrics tools used in analytical chemistry: An overview. Talanta.

[11] Pérez-Marín D, Fearn T, Guerrero JE, Garrido-Varo A (2010). Robustness in pig fat NIRS calibrations by orthogonal projection. Chemom. Intell. Lab. Syst..

[12] Meza-Márquez OG, Gallardo-Velázquez T, Osorio-Revilla G (2010). Application of mid-infrared spectroscopy with multivariate analysis and soft independent modeling of class analogies (SIMCA) for the detection of adulterants in minced beef. Meat Sci..

[13] Cozzolino D, Murray I (2002). Effect of sample presentation and animal muscle species on the analysis of meat by near infrared reflectance spectroscopy. J. Near Infrared Spectrosc..

[14] Prieto N, Andrés S, Giráldez FJ, Mantecón AR, Lavín P (2006). Potential use of near infrared reflectance spectroscopy (NIRS) for the estimation of chemical composition of oxen meat samples. Meat Sci..

[15] Cáceres-Nevado JM, Garrido-Varo A, De Pedro-Sanz E, Pérez-Marín DC (2019). Fourier transform near-infrared spectroscopy coupled to a long fibre optic head for the quality control of IBERIAN pork loins: Intact versus minced. Meat Sci..

[16] Zamora-Rojas E, Pérez-Marín D, De Pedro-Sanz E, Guerrero-Ginel JE, Garrido-Varo A (2012). In-situ Iberian pig carcass classification using a micro-electro-mechanical system (MEMS)-based near infrared (NIR) spectrometer. Meat Sci..

[17] Shackelford SD, Wheeler TL, King DA, Koohmaraie M (2012). Field testing of a system for online classification of beef carcasses for longissimus tenderness using visible and near-infrared reflectance spectroscopy. J. Anim. Sci..

[18] Prieto N, Roehe R, Lavín P, Batten G, Andrés S (2009). Application of near infrared reflectance spectroscopy to predict meat and meat products quality: A review. Meat Sci..

[19] Fernández-Barroso MÁ (2021). Use of NIRS for the assessment of meat quality traits in open-air free-range Iberian pigs. J. Food Compos. Anal..

[20] Arce L (2009). Feasibility study on the use of infrared spectroscopy for the direct authentication of Iberian pig fattening diet. Anal. Chim. Acta.

[21] García-Rey RM, García-Olmo J, De Pedro E, Quiles-Zafra R, Luque de Castro MD (2005). Prediction of texture and colour of dry-cured ham by visible and near infrared spectroscopy using a fiber optic probe. Meat Sci..

[22] Ortiz MC, Sarabia L, García-Rey R, de Castro MDL (2006). Sensitivity and specificity of PLS-class modelling for five sensory characteristics of dry-cured ham using visible and near infrared spectroscopy. Anal. Chim. Acta.

[23] Fernández-Cabanás VM, Polvillo O, Rodríguez-Acuña R, Botella B, Horcada A (2011). Rapid determination of the fatty acid profile in pork dry-cured sausages by NIR spectroscopy. Food Chem..

[24] Prevolnik Povše M (2017). Accuracy of near infrared spectroscopy to predict quality of pork and pork products including samples of Krškopolje and Turopolje pigs. Agric. Conspec. Sci..

[25] García-Olmo J, Garrido-Varo A, De Pedro E (2001). The transfer of fatty acid calibration equations using four sets of unsealed liquid standardisation samples. J. Near Infrared Spectrosc..

[26] Gjerlaug-Enger E, Kongsro J, Aass L, Ødegård J, Vangen O (2011). Prediction of fat quality in pig carcasses by near-infrared spectroscopy. Animal.

[27] Zamora-Rojas E, Garrido-Varo A, De Pedro-Sanz E, Guerrero-Ginel JE, Pérez-Marín D (2013). Prediction of fatty acids content in pig adipose tissue by near infrared spectroscopy: At-line versus in-situ analysis. Meat Sci..

[28] Lebret B (2008). Effects of feeding and rearing systems on growth, carcass composition and meat quality in pigs. Animal.

[29] Conzen, J. P. Multivariate calibration. in *Optik, Bruker. vol. A Practical Guide for Developing Methods in the Quantitative Analytical Chemistry* (2014).

[30] Park BC, Chen YR, Hruschka WR, Shackelford SD, Koohmaraie M (2001). Principal component regression of near- infrared reflectance spectra for beef tenderness prediction. Trans. ASAE.

[31] Peris-Díaz MD, Krężel A (2021). A guide to good practice in chemometric methods for vibrational spectroscopy, electrochemistry, and hyphenated mass spectrometry. TrAC, Trends Anal. Chem..

[32] Giaretta E (2019). NIRs calibration models for chemical composition and fatty acid families of raw and freeze-dried beef: A comparison. J. Food Compos. Anal..

[33] ElMasry G, Nakauchi S (2016). Prediction of meat spectral patterns based on optical properties and concentrations of the major constituents. Food Sci. Nutr..

[34] Wang P, Rajian JR, Cheng J-X (2013). Spectroscopic imaging of deep tissue through photoacoustic detection of molecular vibration. J. Phys. Chem. Lett..

[35] González-Martín I, González-Pérez C, Alvarez-García N, González-Cabrera JM (2005). On-line determination of fatty acid composition in intramuscular fat of Iberian pork loin by NIRs with a remote reflectance fibre optic probe. Meat Sci..

[36] Pieszczek L, Czarnik-Matusewicz H, Daszykowski M (2018). Identification of ground meat species using near-infrared spectroscopy and class modeling techniques—Aspects of optimization and validation using a one-class classification model. Meat Sci..

[37] Garrido-Varo A, Carrete R, Fernández-Cabanás V (1998). Use of difference near infrared reflectance spectra to extract relevant information from the spectra of agro-food products. J. Near Infrared Spectrosc..

[38] Tsai CL, Chen JC, Wang WJ (2001). Near-infrared absorption property of biological soft tissue constituents. J. Med. Biol. Eng..

[39] Pérez-Marín D, De Pedro Sanz E, Guerrero-Ginel JE, Garrido-Varo A (2009). A feasibility study on the use of near-infrared spectroscopy for prediction of the fatty acid profile in live Iberian pigs and carcasses. Meat Sci..

[40] Müller M, Scheeder MRL (2008). Determination of fatty acid composition and consistency of raw pig fat with near infrared spectroscopy. J. Near Infrared Spectrosc..

[41] Oliveri P, Malegori C, Simonetti R, Casale M (2019). The impact of signal pre-processing on the final interpretation of analytical outcomes—A tutorial. Anal. Chim. Acta.

[42] Cozzolino D, Murray I, Scaife JR, Paterson R (2000). Study of dissected lamb muscles by visible and near infrared reflectance spectroscopy for composition assessment. Anim. Sci..

[43] Fan Y, Liao Y, Cheng F (2018). Predicting of intramuscular fat content in pork using near infrared spectroscopy and multivariate analysis. Int. J. Food Prop..

[44] Pugliese C, Sirtori F (2012). Quality of meat and meat products produced from southern European pig breeds. Meat Sci..

[45] Parrini S, Acciaioli A, Franci O, Pugliese C, Bozzi R (2019). Near infrared spectroscopy technology for prediction of chemical composition of natural fresh pastures. J. Appl. Anim. Res..

[46] Prieto N (2011). Online prediction of fatty acid profiles in crossbred Limousin and Aberdeen Angus beef cattle using near infrared reflectance spectroscopy. Animal.

[47] Pérez-Marín D, Garrido-Varo A, De Pedro E, Guerrero-Ginel JE (2007). Chemometric utilities to achieve robustness in liquid NIRS calibrations: Application to pig fat analysis. Chemom. Intell. Lab. Syst..

[48] Flåtten A, Bryhni EA, Kohler A, Egelandsdal B, Isaksson T (2005). Determination of C22:5 and C22:6 marine fatty acids in pork fat with Fourier transform mid-infrared spectroscopy. Meat Sci..

[49] Millmier A (2000). Near-infrared sensing of manure nutrients. Trans. Am. Soc. Agric. Eng..

[50] Williams PC, Norris K (2001). Near-Infrared Technology in the Agricultural and Food Industries.

[51] Williams PC, Sobering DC (1993). Comparison of commercial near infrared transmittance and reflectance instruments for analysis of whole grains and seeds. J. Near Infrared Spectrosc. JNIRS.

[52] Williams P (2014). The RPD statistic: A tutorial note. NIR news.

[53] Folch J, Lees M, Sloane Stanley GH (1957). A simple method for the isolation and purification of total lipides from animal tissues. J Biol Chem.

[54] Morrison WR, Smith L (1964). Preparation of fatty acid methyl esters and dimethylacetals from lipids with boron fluoride–methanol. J. Lipid Res..

[55] Shenk JS, Westerhaus MO (1995). Analysis of Agriculture and Food Products by Near Infrared Reflectance Spectroscopy, Monograph.

[56] Saeys W, Darius P, Ramon H (2004). Potential for on-site analysis of hog manure using a visual and near infrared diode array reflectance spectrometer. J. Near Infrared Spectrosc..

