# Supplementary figures and images for: Prediction of fatty acid composition in intact and minced fat of European autochthonous pigs breeds by near infrared spectroscopy
Source: Sci Rep. 2023 May 15;13:7874. doi: 10.1038/s41598-023-34996-x (PMC10185696; doi:10.1038/s41598-023-34996-x)

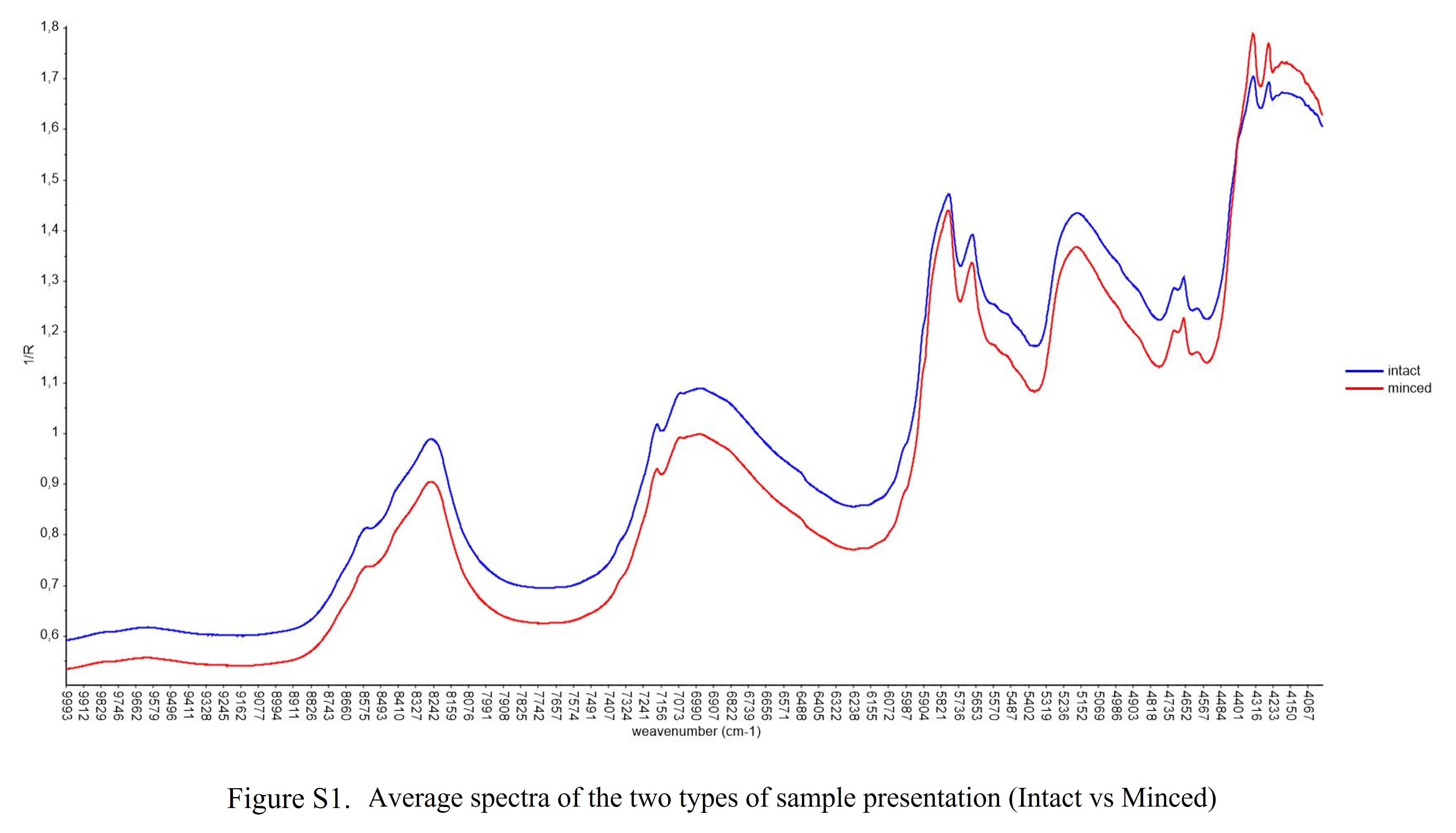

Supplement: Supplementary file 1 — Supplementary Information 1. [file 41598_2023_34996_MOESM1_ESM.jpg]

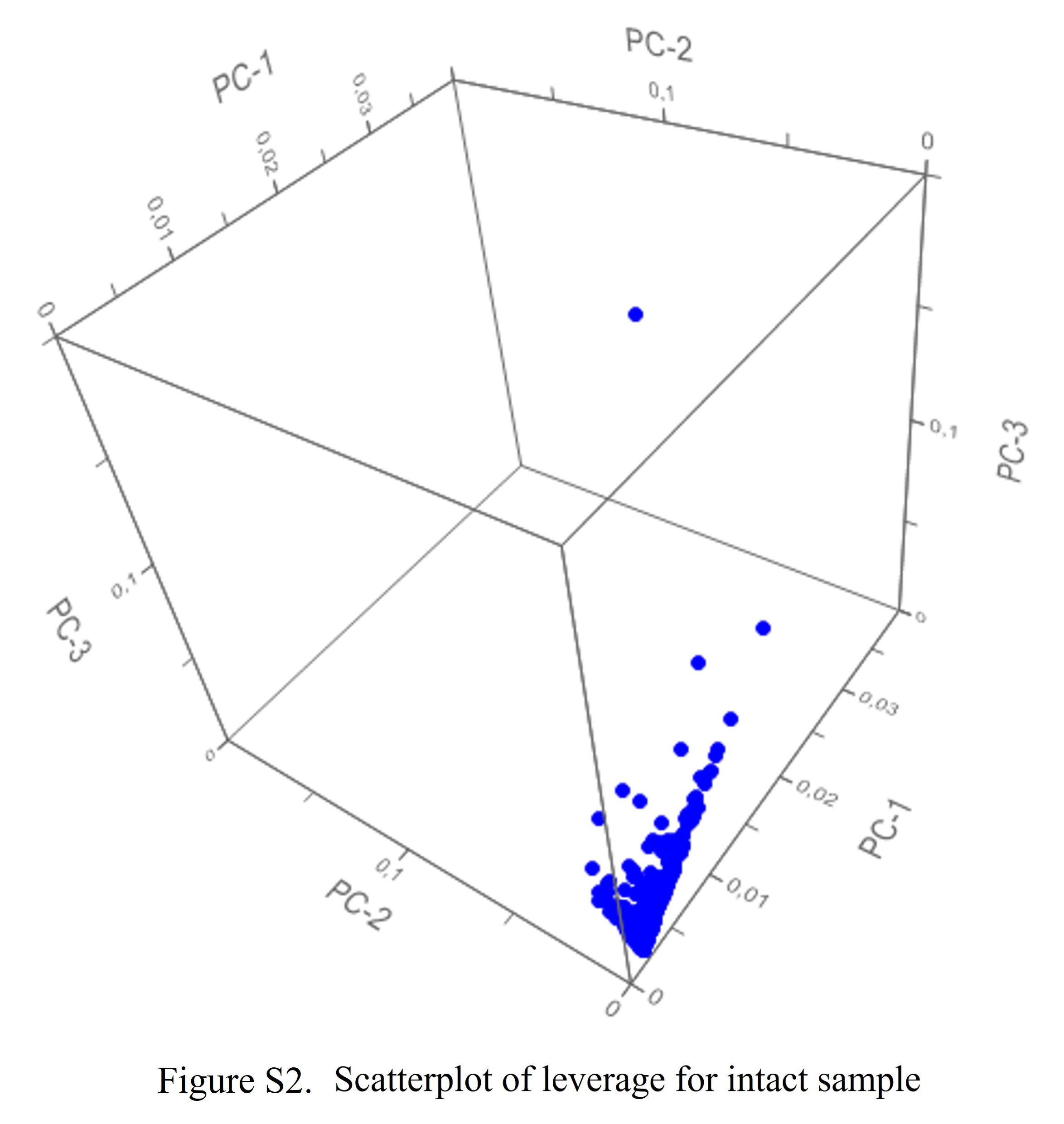

Supplement: Supplementary file 2 — Supplementary Information 2. [file 41598_2023_34996_MOESM2_ESM.jpg]

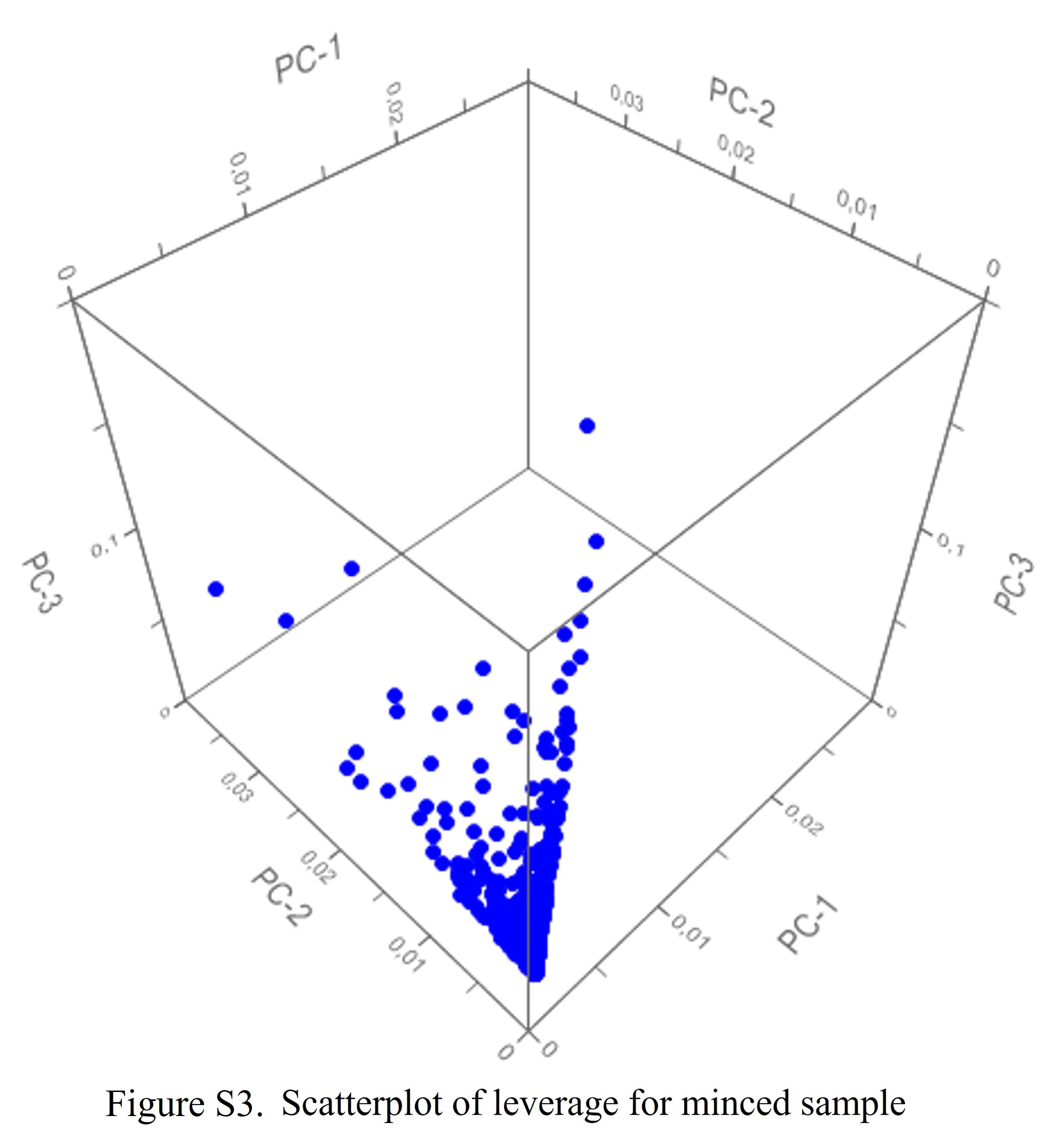

Supplement: Supplementary file 3 — Supplementary Information 3. [file 41598_2023_34996_MOESM3_ESM.jpg]
